# Prognostic value of PD-L1 expression in tumor infiltrating immune cells in cancers: A meta-analysis

**DOI:** 10.1371/journal.pone.0176822

**Published:** 2017-04-28

**Authors:** Tiancheng Zhao, Changfeng Li, Yanhua Wu, Bingjin Li, Bin Zhang

**Affiliations:** 1 Department of Endoscopy Center, China-Japan Union Hospital of Jilin University, Changchun, Jilin, China; 2 Division of Clinical Research, First Hospital of Jilin University, Changchun, Jilin, China; 3 Jilin Provincial Key Laboratory on Molecular and Chemical Genetics, The Second Hospital of Jilin University, Changchun, Jilin, China; Nanjing Normal University, CHINA

## Abstract

Programmed death-ligand 1 (PD-L1) is a promising target of cancer immune therapy. It not only expressed in tumor cells (TCs) but also up regulated in tumor infiltrating immune cells (TIICs). Although the previous meta-analysis have shown that PD-L1 expression in TCs was a valuable biomarker in predicting cancer prognosis, but few researches systematic evaluated the association between its expression in TIICs and survival of cancer patients. Thus, we performed this meta-analysis to evaluate the prognostic value of PD-L1 expression in TIICs in different types of cancers. Our results are valuable supplements when using PD-L1 expression to predict the survival of cancer patients and to select the beneficial patients from PD-L1 target therapy. PubMed, Embase, Web of Science and the Cochrane Central Search Library were used to perform our systematic literature search. Overall survival (OS) at 5th years and hazard ratios (HRs) were calculated using random effects models. Eighteen studies involving 3674 patients were included. The median positive rate of PD-L1 staining in TIICs was 36.37%. PD-L1 positive expression in TIICs related to a lower risk of death (HR = 0.784, 95%CI: 0.616–0.997, *P* = 0.047). Subgroup analyses found that PD-L1 positive expression in TIICs indicated a better prognosis especially in breast cancer patients (HR = 0.359, *P* = 0.041). When using whole tissue section slides, or using ‘any expression in TIICs’ as a cutoff value to assessing the results of IHC staining, PD-L1 expression in TIICs had a good prognostic value in cancer prognosis (HR = 0.587, *P* = 0.001 and HR = 0.549, *P* = 0.002). Our findings suggested that PD-L1 expression in TIICs was related to a better survival of cancer. The comprehensive evaluation of tumor cells and tumor infiltrating immune cells are required when evaluating the effect of PD-L1 expression on prognosis of cancer in future research.

## Introduction

Cancer remains the most prominent global health-related threat[1, 2]. Traditional therapies such as tumorectomy, radiotherapy and chemotherapy are still the main treatments, but their efficacies are unsatisfactory in most cancers, especially in advanced cancers[3]. Recently, variable new cancer treatments have emerged, with immune checkpoint inhibition being one of the most promising therapies[4, 5].

Among the immune checkpoint molecules, programmed death 1 (PD-1) and its ligand, programmed death-ligand 1 (PD-L1), constitute a pair of negative co-stimulatory molecules that can suppress the functions of T cells and mediate the immune escape of cancers[6, 7]. PD-1 and PD-L1 inhibitors were developed by numerous pharmaceuticals companies and well studied in several clinical trials[8, 9]. A meta-analysis including 20 trials reported that patients with positive PD-L1 expression might have a decreased risk of mortality compared to negative cases when treated with anti PD-1/PD-L1 antibodies[10]. And the expression of PD-L1 not only linked to the response of immune checkpoint therapy but also associated with the prognosis of several types of cancer, such as non-small-cell lung cancer[11], gastric cancer[12], and breast cancer[13].

Although there has been already a lot of literatures published investigated the associations between PD-L1 expression and cancer prognosis using the method of meta-analysis[14]. However, all of them focused only on the PD-L1 expression in tumor cells. As we know, PD-1/PD-L1 pathway plays an important role in the cancer-specific immune response. PD-L1 is not only expressed in tumor cells but also up-regulated in tumor infiltrating immune cells (TIICs), including tumor infiltrating lymphocytes, mononuclear cells and other immune cells[15–17]. Current clinical trials have shown that the expression of PD-L1 in TIICs is also indicative of a higher response rate to PD-L1/PD-1 targeted therapy[18, 19]. Therefore, not only tumor cell-based but also immune cell-based PD-L1 expression appears to be clinically valuable. Recently, a number of studies have reported that the expression of PD-L1 in TIICs which was correlated with the survival of patients with tumors, but have failed to reach consistent conclusions [20–26]. In addition, there has been no research systematic evaluates the predicted value of PD-L1 positive expression in TIICs in cancer prognosis.

Thus, we performed this meta-analysis to evaluate the prognostic value of PD-L1 expression in TIICs in different types of cancers. Our results are valuable supplements when using PD-L1 expression to predict the survival of cancer patients and to select the beneficial patients from PD-L1 target therapy.

## Materials and methods

### Search strategy

PubMed, Embase, Web of Science and the Cochrane Central Search Library were used to perform our systematic literature search (until December 2016). Key words used included “programmed death-ligand 1 or PD-L1 or B7-H1 or CD274” and “tumor infiltrating lymphocyte or TIL or tumor infiltrating immune cells or TIIC or tumor infiltrating mononuclear cells or TIMC or tumor stroma” and “cancer or carcinoma or tumor” and “prognosis or survival”; the results were limited to human studies. In addition, we searched the reference lists of the reviews on related topics by hand to identify additional studies.

### Inclusion and exclusion criteria

The eligible studies were included in this meta-analysis based on the following criteria: (1) PD-L1 expression has been measured by immunohistochemistry (IHC) stain in tumor infiltrating immune cells rather than in tumor cells; (2) studies reported 5-year OS, HR with 95% confidence interval (95% CIs), or reported original survival curves; (3) studies were published in English, and their full texts were available. Exclusion criteria for this study were as follows: (1) conference abstracts, letters, reviews and unpublished studies; and (2) insufficient data to report the hazard ratios and 95% CI, or could not extract the data from Kaplan-Meier curves. If duplicate data presented in more than one study, the largest or most recent study was included.

### Data extraction and quality assessment

Two reviewers (Zhao TC and Wu YH) identified relevant articles independently. The details of these surveys included the author’s name, date of publication, type of cancer, type of pathological section, number of patients, tumor stage, age of patients, duration of follow up, antibody, staining location, and cut-off value in assessing the positive expression of PD-L1 in tumor infiltrating immune cells. Newcastle-Ottawa Scale (NOS) was used for quality assessment[27]. NOS scores no less than 6 were defined as high-quality studies.

### Statistical analysis

Data were analyzed using STATA version 12.0 (STATA Corporation, College Station, TX, USA). The 3-year OS, 5-year OS, HR and 95%CI were extracted from the original studies, tables or recalculated from Kaplan—Meier curves using the program of Engauge Digitizer (http://sourceforge.net/projects/digitizer/), and applied using the Mantel—Haenszel random effect model. I^2^[28] was used to determine the heterogeneity of the selected studies. Subgroup analysis and sensitivity analysis were carrying out to assess the potential contributions of different type of tumors and different cutoff values for defining PD-L1 expression in TIICs. Egger’s regression asymmetry test[29] and Begg’s adjusted rank correlation[30] were performed to evaluate the potential publication bias. All tests were two-sided, and *P*<0.05 was considered statistically significant.

## Results

### Identification of eligible studies

**Fig 1:**
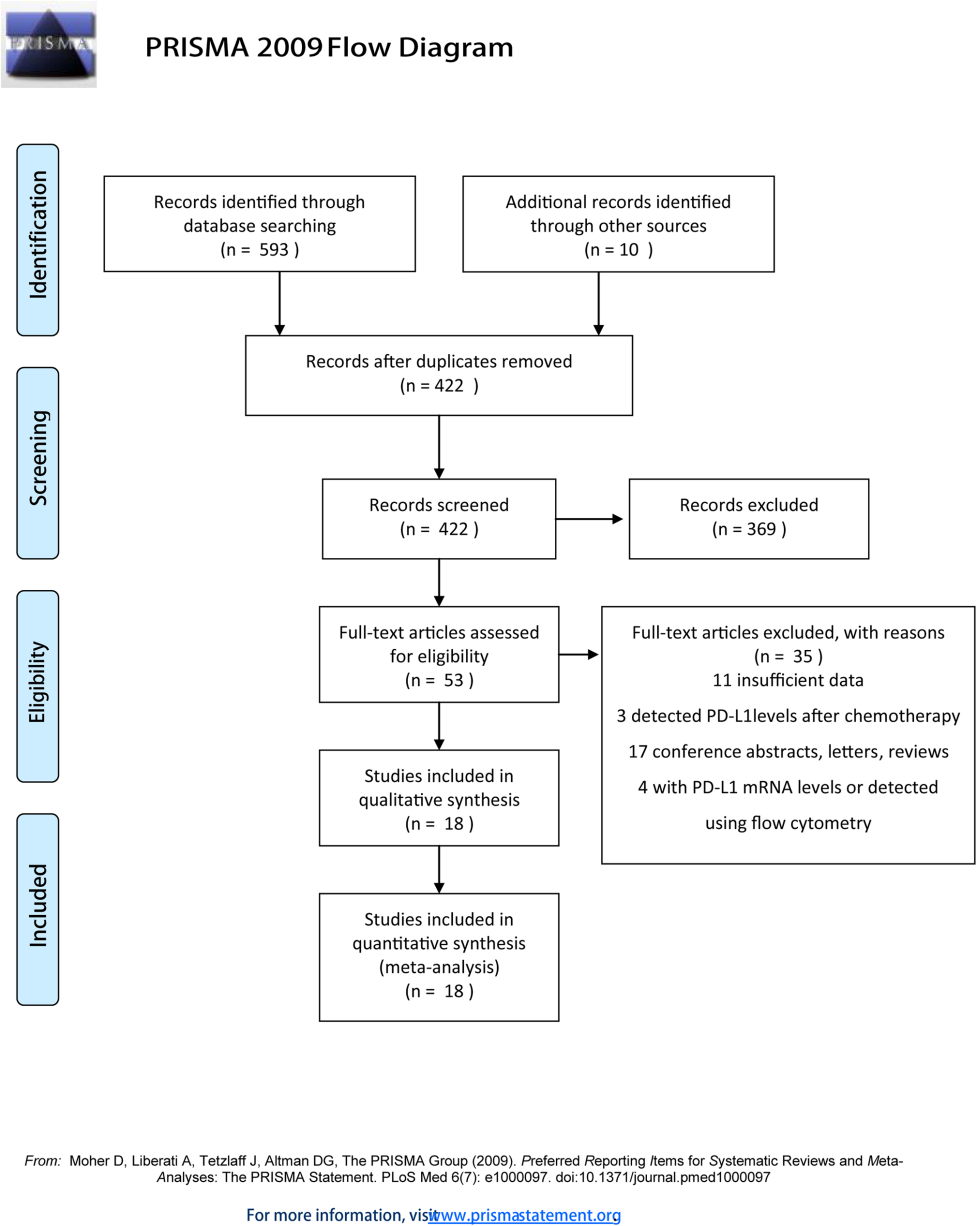
Flow diagram of the literature search and study selection for the meta-analysis. The flow diagram shows eligible publications at each stage of the analysis process. The database search was conducted in December 2016.

The search results shown in Fig 1 identified 603 studies from the initial database. After careful manual selection and review of these articles, 18 studies with full text and available data according to the inclusion and exclusion criteria were included in the final analysis (Fig 1). And the PRISMA checklist was showed in S1 File.

### Description of studies

The characteristics of the 18 studies summarized in Table 1. All of the studies assessed PD-L1 expression in tumor infiltrating immune cells using immunohistochemistry techniques. Seven studies evaluated cancers of the digestive system (5 gastric cancers, 1 esophageal squamous cell carcinoma and 1 colorectal cancer), two evaluated cancers of the urinary system (1 urothelial carcinoma and 1 renal cell carcinoma), two evaluated breast cancer, two evaluated lung cancer, and one each evaluated ovarian high grade serous carcinoma, extranodal NK/T-cell lymphoma, head and neck cancer, diffuse large B-cell lymphoma and spinal chordoma. In total, 3674 subjects were included in our meta-analysis.

**Table 1:** Characteristics of the studies included.

No	Study, Year	Cancer types	Tissue slides	No. of patients	Age (range)	Tumor stages^a^	Follow up (months)	PD-L1 NO (+/-)
1	Bellmunt J,2015[20]	urothelial carcinoma	TMAs	89	NR	IV (metastatic)	1–24	33/56
2	Boger C,2016[21]	gastric cancer	Whole	465	68 (median)	I-IV	0.2–109	160/291
3	Choueiri TK,2014[22]	nonclear-cell renal cell carcinoma	NR	101	24–81	I-IV	60(median)	57/44
4	Darb-Esfahani S,2015[23]	ovarian high grade serous carcinoma	TMAs	200	60 (median)	I-IV (FIGO)	37.9(median)	60/140
5	Hatogai K,2016[24]	esophageal squamous cell carcinoma	TMAs	196	42–87	I-IV	1.2–127.2	119/77
6	Hou J,2014[25]	gastric cancer	NR	111	18–96	I-IV	NR	71/40
7	Jo J-C,2016[26]	extranodal NK/T-cell lymphoma	NR	79	19–79	I-IV	52.4(median)	62/17
8	Kawazoe A,2016[31]	gastric cancer	TMAs	383	26–92	III-IV	NR	241/142
9	Kim HR,2016[32]	head and neck cancer	TMAs	402	22–88	I-IV	46.3 (median)	112/290
10	Kiyasu J,2015[33]	diffuse large B-cell lymphoma	NR	239	30–92	I-IV	NR	53/186
11	Li X,2016[34]	triple-negative breast cancer	Whole	136	NR	NR	NR	32/104
12	Paulsen E-E,2016[35]	none small cell lung cancer	TMAs	505	28–85	I-IIIA	86(34–267)	182/323
13	Saito R,2016[36]	EBV positive gastric cancer	TMAs	96	40–90	I-IV	3-262(range)	43/53
14	Sun W-Y,2016[37]	triple negative breast cancer	TMAs	218	NR	I-IIIB	0.2–98 (range)	80/138
15	Thompson ED, 2016[38]	gastric adenocarcinomas	Whole	33	21–92	I-IV	40(median)	15/18
16	Wang L,2016[39]	Colorectal cancer	TMAs	262	28–75	II-III	43.5(mean) (21–68)	55/207
17	Yang C-Y,2016[40]	pulmonary squamous cell carcinoma	Whole	105	40–84	IA-IB	79(mean)	31/74
18	Zou MX,2016[41]	spinal chordoma	Whole	54	23–79	I-III	42.39(mean) (5–158)	12/42

NR, not reported; TMAs, tissue microarrays; EBV, Epstein-Barr virus; FIGO: International Federation of Gynecology and Obstetrics

^a^ Unless otherwise noted, Tumor stage was classified according to the AJCC/UICC staging system

### Evaluation of PD-L1 expression in TIICs

The antibodies, cutoff values and staining locations used in the evaluation of PD-L1 expression in TIICs of the included studies are shown in Table 2. Clone E1L3N was used in four studies, and Clone SP142 was used in three studies. The cutoff values in assessing the positive expression of PD-L1 in TIICs were divided into 4 types: (1) proportion of stained cells greater than 5%, (2) proportion of stained cells greater than 1%, (3) any expression of PD-L1 in TIICs and (4) others. Most studies considered that the positive staining was located in the cell membrane (12 of 18 studies); whereas others thought both membranous and cytoplasmic staining could be considered as positive expression. The median positive rate of PD-L1 expression in TIICs was 36.37%.

**Table 2:** Detection of the PD-L1 expression in TIICs in the selected studies.

No	Study, Year	Antibody	Cutoff value of PD-L1 positive expression in TIICs	Staining location	3-year OS(+/-)%	5-year OS (+/-)%
1	Bellmunt J,2015 [20]	405.9A11	Absent (0), focal (1), mild (2), moderate (3), and severe (4); 2–4 were considered positive	Membrane	NR	NR
2	Boger C,2016 [21]	E1L3N	The percentage of positive cells: 0 (negative), 1 (1–5% positive), 2 (6–20%) and 3 (>20%); Score >1 were considered positive	Membrane	39.4/18.8	23.8/12.0
3	Choueiri TK,2014 [22]	405.9A11	According to the percentages of PD-L1 positive TIMC (0% = 0, <5% = 1, ≥5% = 2); Score >0 were considered positive	Membrane	84.5/94.9	73.7/84.1
4	Darb-Esfahani S [23]	EPR1161	>20/mm^2^ were considered positive	Membrane/ Cytoplasm	NR	NR
5	Hatogai K,2016 [24]	NR	Any expression of PD-L1 in TIICs in the core were considered positive	Membrane	58.0/40.9	52.9/33.8
6	Hou J,2014 [25]	NR (Abcam)	Proportion of stained cells >5% were considered positive	Membrane/ cytoplasm	42.3/70.0	NR
7	Jo J-C,2016 [26]	NR (R&D Systems)	More than 5% cells was stained were considered positive	Membrane/ cytoplasm	54.3/30.1	48.6/30.1
8	Kawazoe A,2016 [31]	SP142	<1% (0), 1% to 9% (2), 10% to 19% (3), ≥20% (4); ≥1% were considered positive	Membrane	62.9/59.1	55.5/48.5
9	Kim HR,2016 [32]	SP142	Proportion of stained cells >5% were considered positive	Membrane/ cytoplasm	93.7/80.1	90.2/75.5
10	Kiyasu J,2015 [33]	ab174838	PD-L1 nonmalignant stromal cells represented 20% or more of the total tissue were considered positive	Membrane/ cytoplasm	63.3/72.9	51.6/61.1
11	Li X,2016 [34]	E1L3N	Any stromal PD-L1 expression were considered positive	Membrane	NR	NR
12	Paulsen E-E,2016 [35]	E1L3N	Absent (0), 1% to 49% (1), 50% to 75% (2), or > 75% (3) >1.5 were considered positive	Membrane/ cytoplasm	NR	52/44
13	Saito R,2016 [36]	E1L3N	Simply classified into negative or positive groups depending on the proportion of stained cells (cutoff value: 1%)	Membrane	88.0/91.7	80.9/91.7
14	Sun W-Y,2016 [37]	28–8	any immunostaining were considered positive	Membrane	NR	NR
15	Thompson ED, 2016 [38]	5H1	>1% of PD-L1 staining on TIL or TAM was considered positive."	Membrane	71.2/76.0	51.6/61.1
16	Wang L,2016[39]	SP142	<1% (0), 1% to 4% (1), 5% to 9% (2), ≥10% (3); scores of 2 and 3 were considered positive	Membrane	66.5/80.1	57.4/72.5
17	Yang C-Y,2016 [40]	17952–1 -AP	Proportion of stained cells >5% were considered positive	Membrane	NR	NR
18	Zou MX,2016 [41]	ab174838	Absent (0), rare/few (1), moderate (2), prominent (3), ≥2 were considered positive	Membrane	87.6/93.3	72.7/32.5

NR, not reported; TIICs, tumor infiltrating immune cells; TIL, tumor-infiltrating lymphocyte; TAM, tumor-associated macrophages

### PD-L1 expression in TIICs and five-year OS

**Fig 2:**
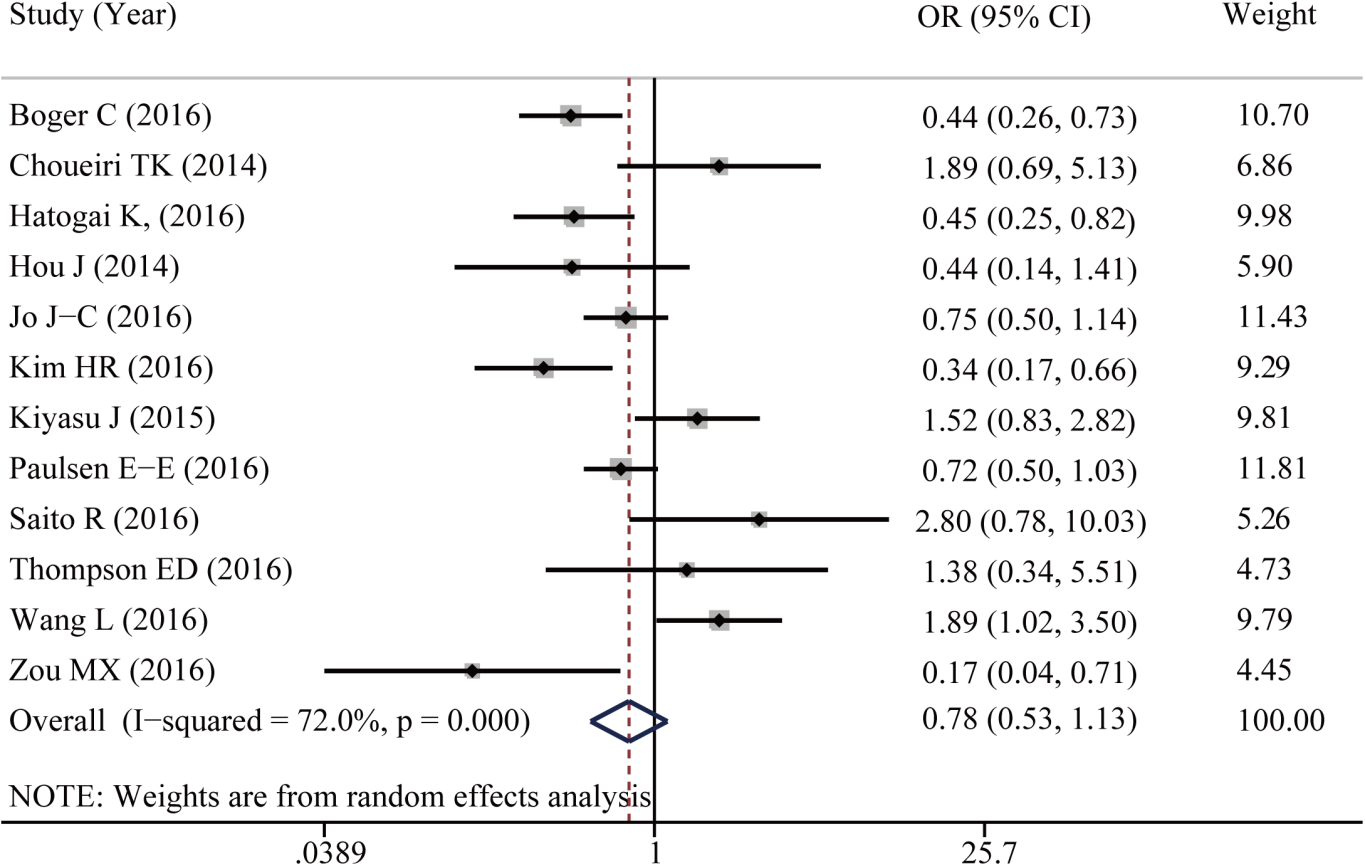
Forest plot shows the associations between PD-L1 expression in TIICs and five year overall survival of cancer patients.

**Fig 3:**
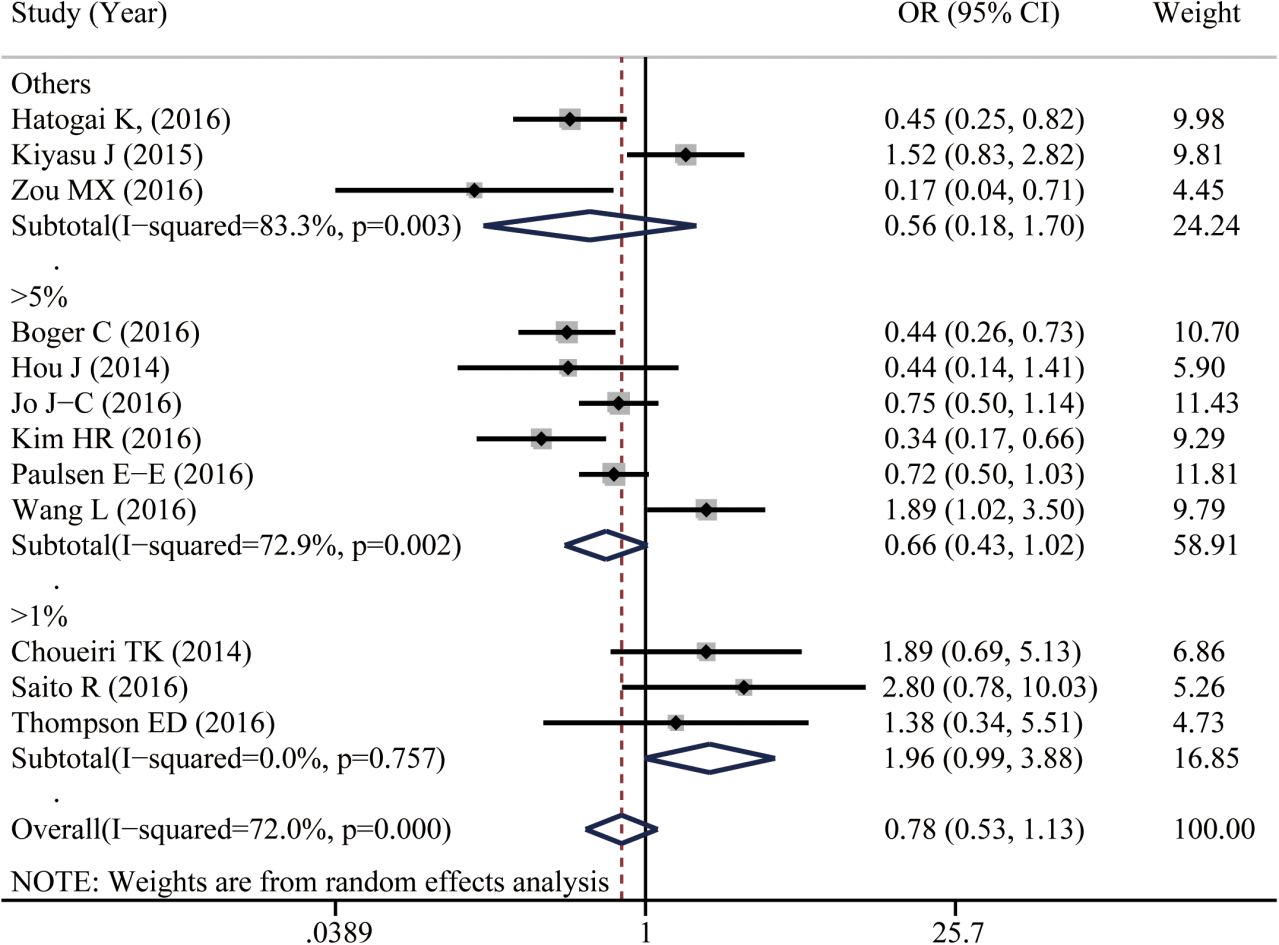
Subgroup analysis by different cutoff values shows the associations between PD-L1 expression in TIICs and five year overall survival of cancer patients.

Twelve studies reported data for 5-year OS. As shown in Fig 2, PD-L1 positive expression in TIICs seems to be associated with a better 5-year OS of cancer patients, though it did not reach statistical difference (OR = 0.778, 95%CI: 0.534–1.134, *P* = 0.192). Because of the significant heterogeneity among studies (I^2^ = 72%), subgroup analyses were conducted to assess whether the heterogeneity was due to different cancer types and cutoff values. Six studies provided the 5-year OS for digestive system cancers; others reported different types of cancer (S1 Fig). In the stratified analysis by cancer types, PD-L1 positive expression in TIICs of digestive system cancers was not associated with 5-year OS (OR = 0.862, 95%CI: 0.438–1.697, *P* = 0.667). Further, I^2^ was calculated to be 76.5%, which indicated that the heterogeneity was not due to different cancer types. We then conducted a subgroup analysis according to different cutoff values. When the cutoff value was defined as ‘proportion of stained cells greater than 5%’, PD-L1 expression in TIICs seems to be associated with a better cancer survival (OR = 0.662, 95% CI: 0.429–1.022, *P* = 0.062, I^2^ = 72.9%). When using the cutoff value of ‘proportion of stained cells greater than 1%’ to distinguish the positive and negative expression of PD-L1 in TIICs, the opposite trend has been reported (OR = 1.958, 95%CI: 0.987–1.134, *P* = 0.055, I^2^ = 0%; Fig 3).

### PD-L1 expression in TIICs and time-to-event index

**Fig 4:**
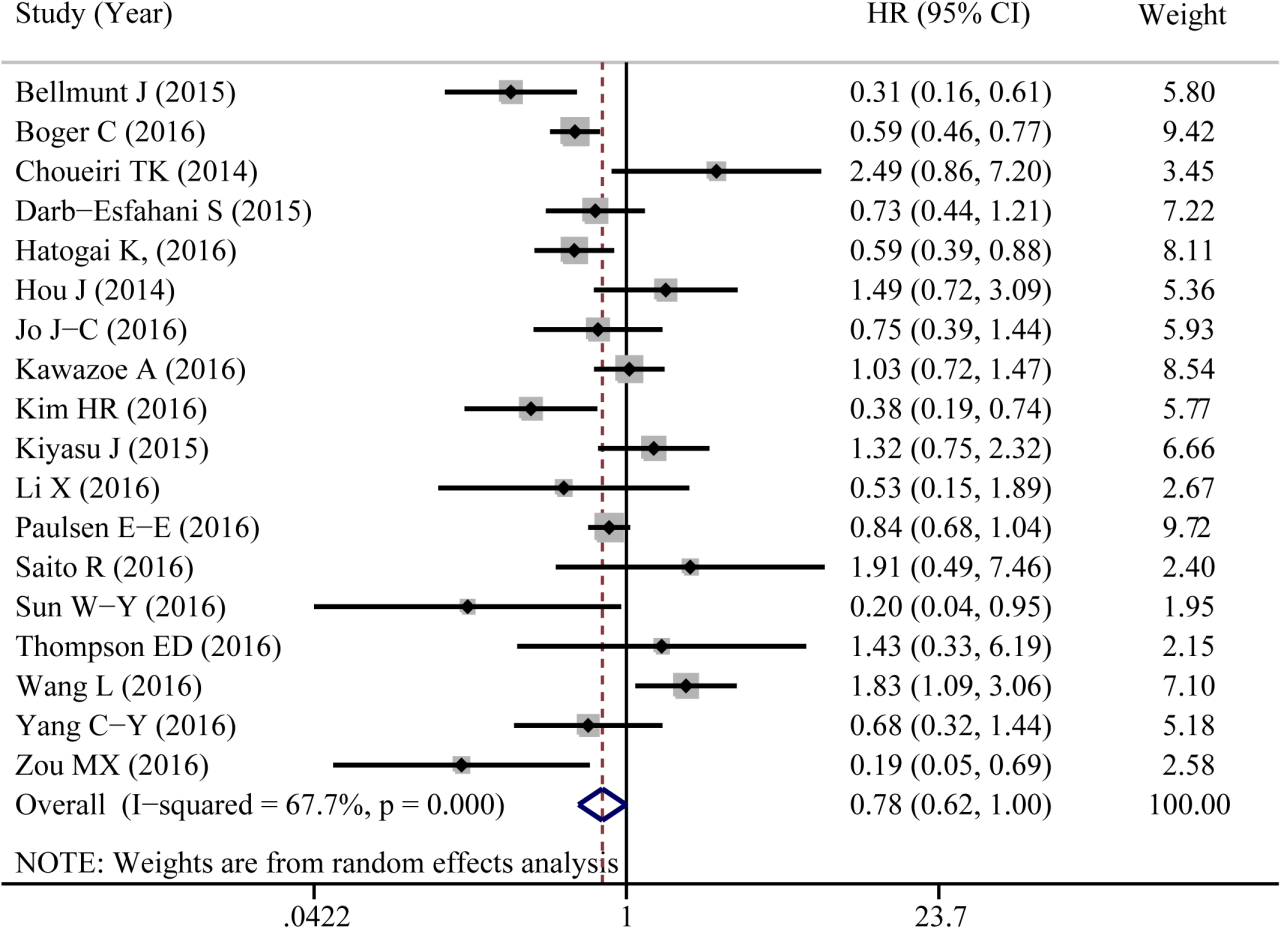
Forest plot of hazard ratios shows the associations between PD-L1 expression in TIICs and cancer prognosis.

**Fig 5:**
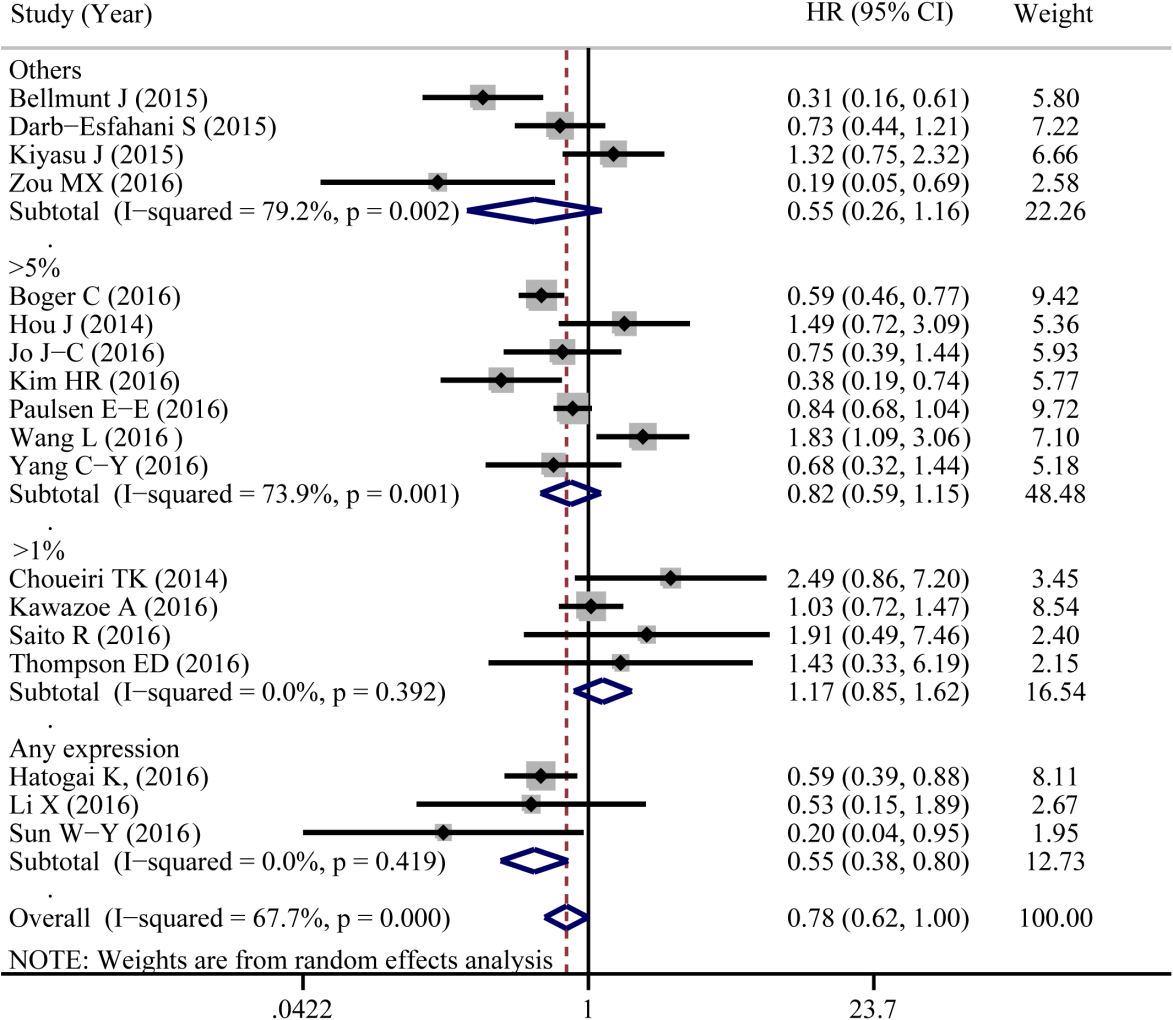
Forest plot of hazard ratios form subgroup analysis by different cutoff values shows the associations between PD-L1 expression in TIICs and cancer prognosis.

**Fig 6:**
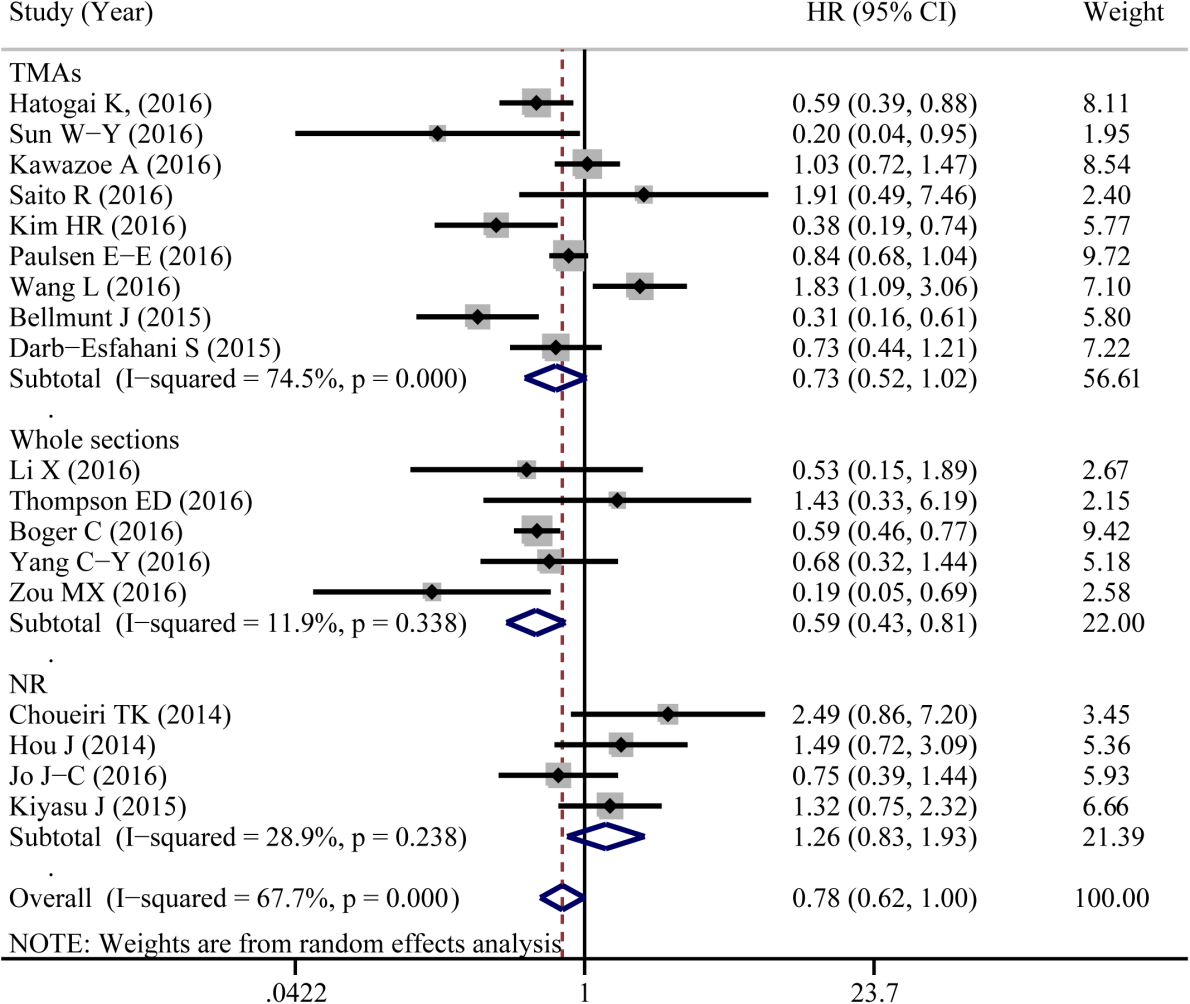
Forest plot of hazard ratios form subgroup analysis by different types of pathological sections shows the associations between PD-L1 expression in TIICs and cancer prognosis. TMAs, tissue microarrays.

**Fig 7:**
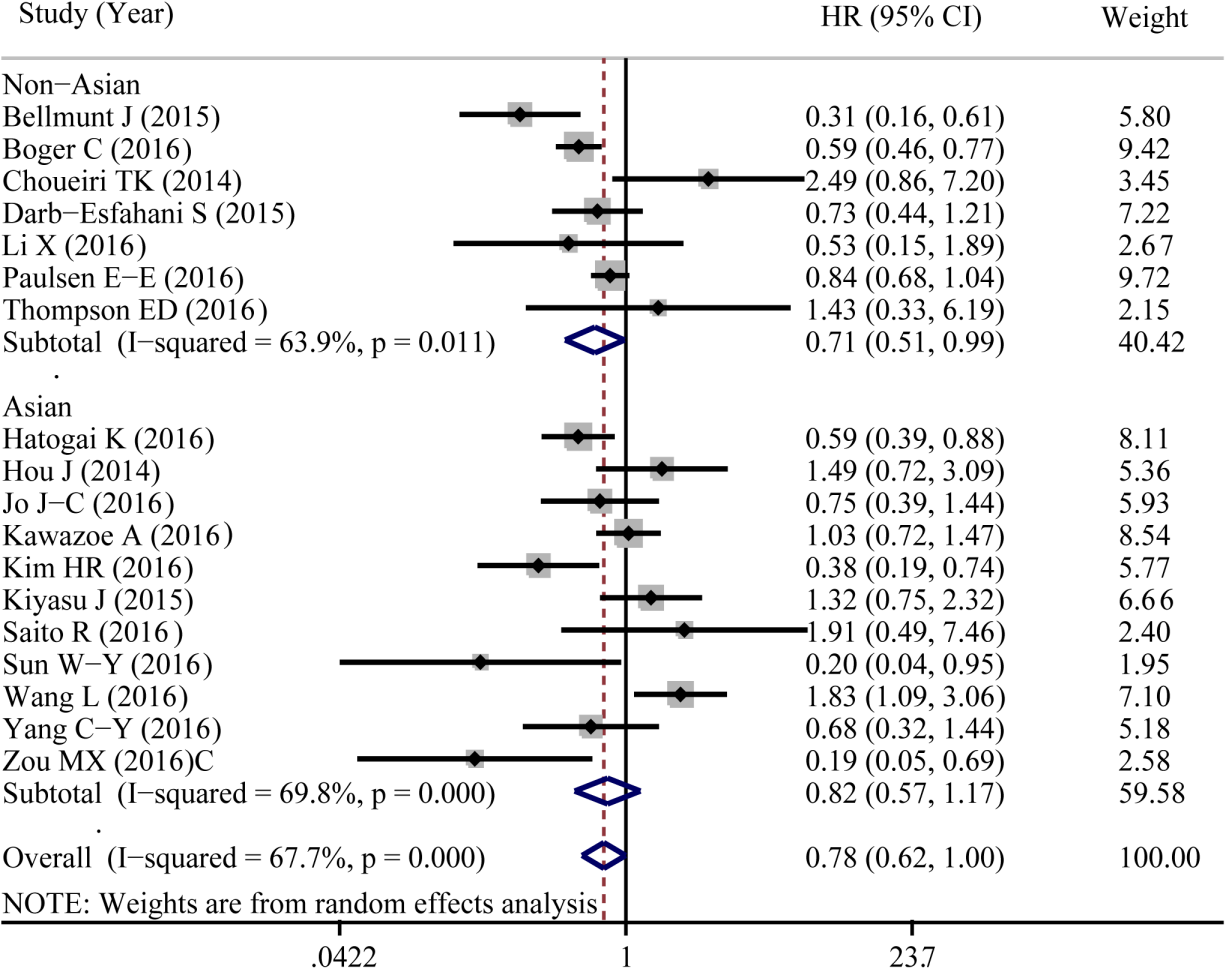
Forest plot of hazard ratios form subgroup analysis by different ethnicity shows the associations between PD-L1 expression in TIICs and cancer prognosis. TMAs, tissue microarrays.

A total of 18 eligible studies were pooled to analyze the predictive value of TIICs expressed PD-L1 in cancer prognosis using HR and 95%CIs. Fig 4 shown that PD-L1 expression in TIICs indicated a decreased risk of death (HR = 0.784, 95%CI: 0.616–0.997, *P* = 0.047). Similar to 5-year OS, significant heterogeneity was noted (I^2^ = 67.7%, *P*<0.001). Exploratory subgroup analysis suggested that PD-L1 expression in TIICs indicted a lower risk of death in patients with breast cancer (HR = 0.359, 95%CI: 0.134–0.961, *P* = 0.041, I^2^ = 0%; S2 Fig). As shown in Fig 5, PD-L1 in TIICs was only associated with improved overall survival in those studies using cutoff value of ‘Any positive staining in immune cells’ (HR = 0.549, 95%CI = 0.378–0.798, *P* = 0.002, I^2^ = 0%). We also conducted a subgroup analysis according to different types of pathological sections (the whole tissue section slides or tissue microarrays). PD-L1 in TIICs was correlated to a favorable prognosis in those studies using whole tissue section slides in conducting the immunohistochemical stain (HR = 0.587, 95%CI: 0.425–0.810, *P* = 0.001, I^2^ = 11.9%, Fig 6). Additionally, genetic differences will contribute to the heterogeneity between individual studies. The ethnicities of included studies were divided into two parts; Asian and non-Asian. Positive expression of PD-L1 in TIICs was an indicator of a favorable prognosis, only in non-Asian cancer patients (HR = 0.709, 95%CI: 0.511–0.985, *P* = 0.040, I^2^ = 63.9%, Fig 7).

### Sensitivity and publication bias analyses

**Fig 8:**
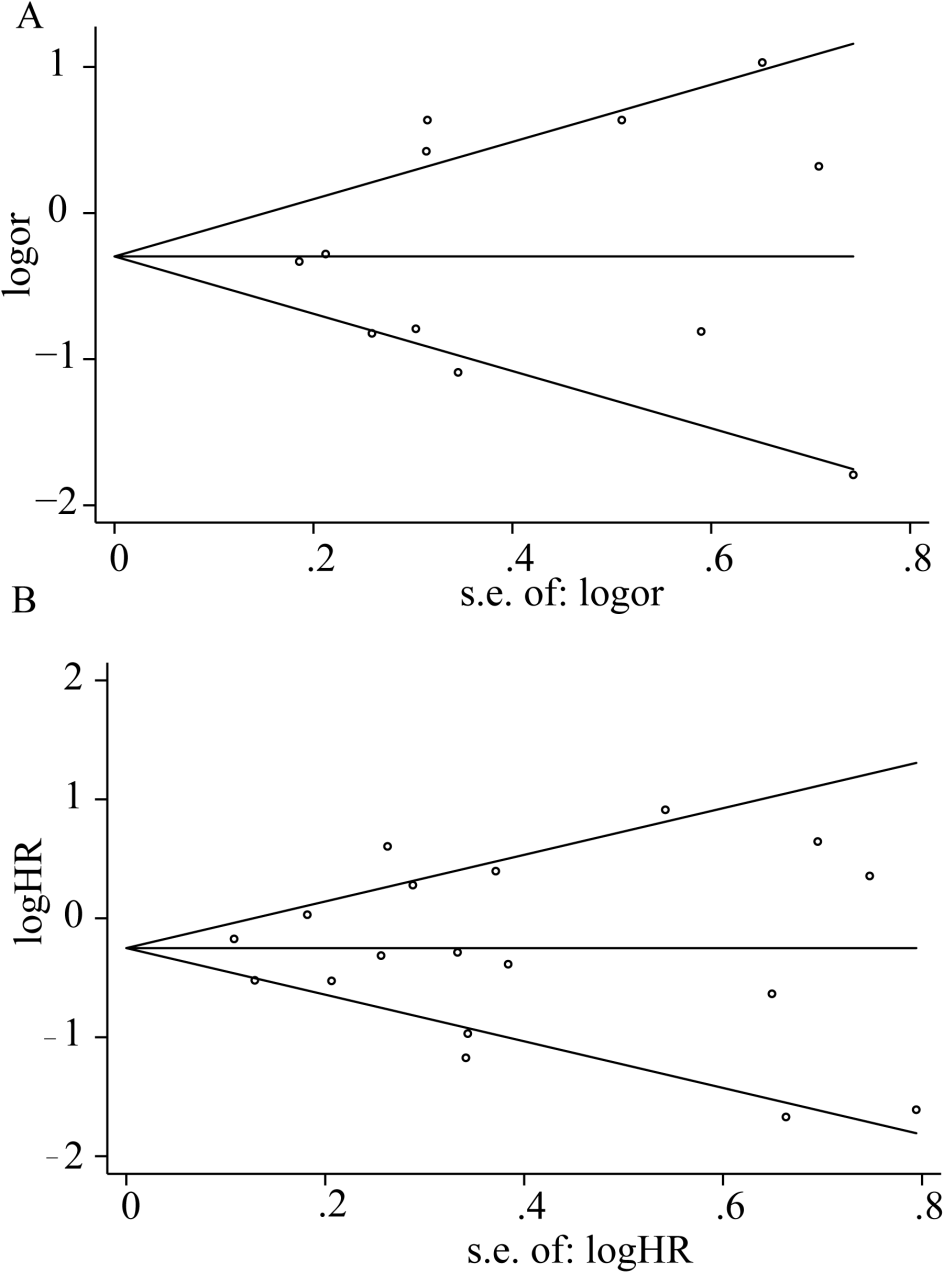
Begg’s funnel plots show the publication bias. (A) Begg’s funnel plot for 5-years OS (B) Begg’s funnel plot for HR.

Omitting any individual study did not influence the combined results for 5-year OS or HR (S3 Fig). The funnel plot for the relationship between PD-L1 expression in TIICs and cancer prognosis is presenting in Fig 8. For 5-year OS the P values for Egger’s and Begg’s tests were 0.714 and 0.891. For hazard ratio, the results from Egger’s and Begg’s tests also revealed that there was no publication bias in this meta-analysis (*P* = 0.986 and *P* = 0.733, respectively).

## Discussion

Anti-PD-1/PD-L1 therapy has been discussed as a potential effective strategy for cancer treatment, and numerous studies have reported the positive expression of PD-L1 in tumor cells as a predictive biomarker for the response to PD-1/PD-L1 blocking therapy[42]. Simultaneously, the expression of PD-L1 has always been considered a predicted biomarker in cancer prognosis[14]. Not only tumor cells, but also tumor-infiltrating immune cells could express PD-L1. Previous studies have only focused on PD-L1 expression in tumor cells, but recent studies indicated that the PD-L1 expression in TIICs also played an important role in tumor immune escape and influenced tumor progression[16, 17, 31, 36]. These leading studies highlighted that PD-L1 expression in TIICs could also serve as a prognostic biomarker, and further inform the responses to anti-PD-1/PD-L1 treatment. In this meta-analysis of data from 18 studies, with a cohort of 3674 cancer patients, we firstly provided a quantitative estimate to the prognostic value of PD-L1 expression in TIICs in cancer patients.

Five-year overall survival and hazard ratio were both important indexes in cancer prognostic evaluation. Our results shown that, PD-L1 in TIICs was significantly associated with a decreased risk of death (HR = 0.784, 95%CI: 0.616–0.997, *P* = 0.047) compared with patients with PD-L1 negative expression in TIICs. A similar trend has observed when using 5-year OS to evaluate the predictive value of PD-L1 in TIICs in cancer prognosis, but the P value was not statistically significant. Our results were quite different from the published meta-analysis which shown that PD-L1 expression in tumor cells was associated with a worse prognosis of cancer. Different mechanisms between PD-L1 expression in TCs and TIICs might explain the inconsistent results. Transcriptome analyses indicated that, PD-L1 expression in TCs was up regulated through the tumor-intrinsic mechanisms, including the activation of endogenous oncogene and related signaling pathway[43]. However, PD-L1 expression in TIICs could be driven by adaptive mechanisms such like exogenous inflammation mediated immune attack and then reflected pre-existing immunity[44, 45]. In other words, comparing with tumor cells, the tumor infiltrating immune cells based PD-L1 expression has stronger relations with cancer immune response, and depends on tumor microenvironments. In fact, PD-L1 positive expression in TIICs was positive correlated to the quantity of multiple tumor-infiltrating immune cells, such as CD4^+^ T lymphocytes and CD8^+^T lymphocytes. Since the high expression of CD4^+^ T lymphocytes and CD8^+^ T lymphocytes was associated with better outcomes of cancer patients[43, 46], the PD-L1 expression in TIICs was possibly associated with better cancer prognosis. Although PD-L1 expression could mediate the occurrence of cancer immune escape, it also indicated an effective immune response, especially with a favorable profile of immune microenvironments in the early stage of the cancer immune response[40].

According to the results of subgroup analysis, different types of pathological sections and different definition of cutoff values when conducting and assessing the IHC stain could partly explain the large heterogeneity among individual studies. Half of the studies using tissue microarrays (TMAs) to conduct the IHC stain. TMAs usually contain limited tissue (2.0mm) and extract from the central part of tumors. Compare with the whole tissue section slides, TMAs may have less representativeness, especially in assessing the biomarkers expressed in tumor infiltrating immune cells. The subgroup analysis according to different types of pathological sections showed that when using whole section slides to investigate PD-L1 positive expression in TIICs, the heterogeneity among different studies was the lowest. It indicates that using whole section slides to conduct the IHC staining should be recommended in related clinical trials and treatments. Additionally, the appropriate cutoff value in validating the positive expression of PD-L1 remains contentious. Subgroup analysis with different cutoff values has shown that there was a contradictory trend when using the cutoff value of ‘5%’ or ‘1%’ in evaluating the correlations of PD-L1 positive expression in TIICs with survival of cancer patients. Therefore, a multi-classification of cutoff values for assessing PD-L1 expression in TIICs may be feasible and reasonable. Asian and non-Asian cancer patients exhibit distinct tumor immunity signatures. For instance, in gastric cancer, non-Asian patients show significantly higher expression level of T-cell markers, including CD3 and CD8, and lower expression level of immunosuppressive T-regulatory cell markers, such as FOXP3 compared to Asian gastric patients[47]. Immune-related biomarkers differentially expressed between Asian and non-Asian cancer patients who was related to immune function. These differences may affect the associations between PD-L1 expression and survival of cancer patients.

Several limitations should be acknowledged in our study. First, in several studies, when 5-year OS and HRs not provided in the original studies, we derived the indexes from Kaplan–Meier survival curves; six of the 18 studies did not provide 5-year OS or original Kaplan–Meier survival curves. As a result, only 12 studies were available to calculate the association of PD-L1 expression in TIICs with five-year OS, which could affect the level of evidence. Second, not all of the included studies using the multiple Cox regression to estimate the independent prognostic value of PD-L1 expression in TIICs in cancer. Thus, the results from all of the studies could not be further stratified with the same confounding factors, and further studies with more confounding factor adjustments need to be conducted.

## Conclusions

Despite these limitations, we have demonstrated that PD-L1 expression in TIICs might serve as a new biomarker for prognosticating the survival of cancer patients. Thus, incorporating the expression of tumor-infiltrating immune cells into the classification of PD-L1 expression is necessary. Our results may be useful supplements when using PD-L1 expression to predict the survival of cancer patients and to select the beneficial patients from anti-PD-L1 treatment.

## Supporting information

S1 Fig Subgroup analysis by different types of cancers shows the associations between PD-L1 expression in TIICs and five year overall survival of cancer patients. (TIF)

S2 Fig Forest plot of hazard ratios form subgroup analysis by types of cancers shows the associations between PD-L1 expression in TIICs and cancer prognosis. (TIF)

S3 Fig Sensitivity analyses show the associations between PD-L1 expression in TIICs and cancer prognosis. (A) Sensitivity analysis for 5-years OS (B) Sensitivity analysis for HR.
(TIF)

S1 File PRISMA 2009 checklist. (DOC)

S2 File Full electronic search strategy in PUBMED. (DOCX)
