# Supplementary figures and images for: Prognostic value of PD-L1 expression in tumor infiltrating immune cells in cancers: A meta-analysis
Source: PLoS One. 2017 Apr 28;12(4):e0176822. doi: 10.1371/journal.pone.0176822 (PMC5409185; doi:10.1371/journal.pone.0176822)

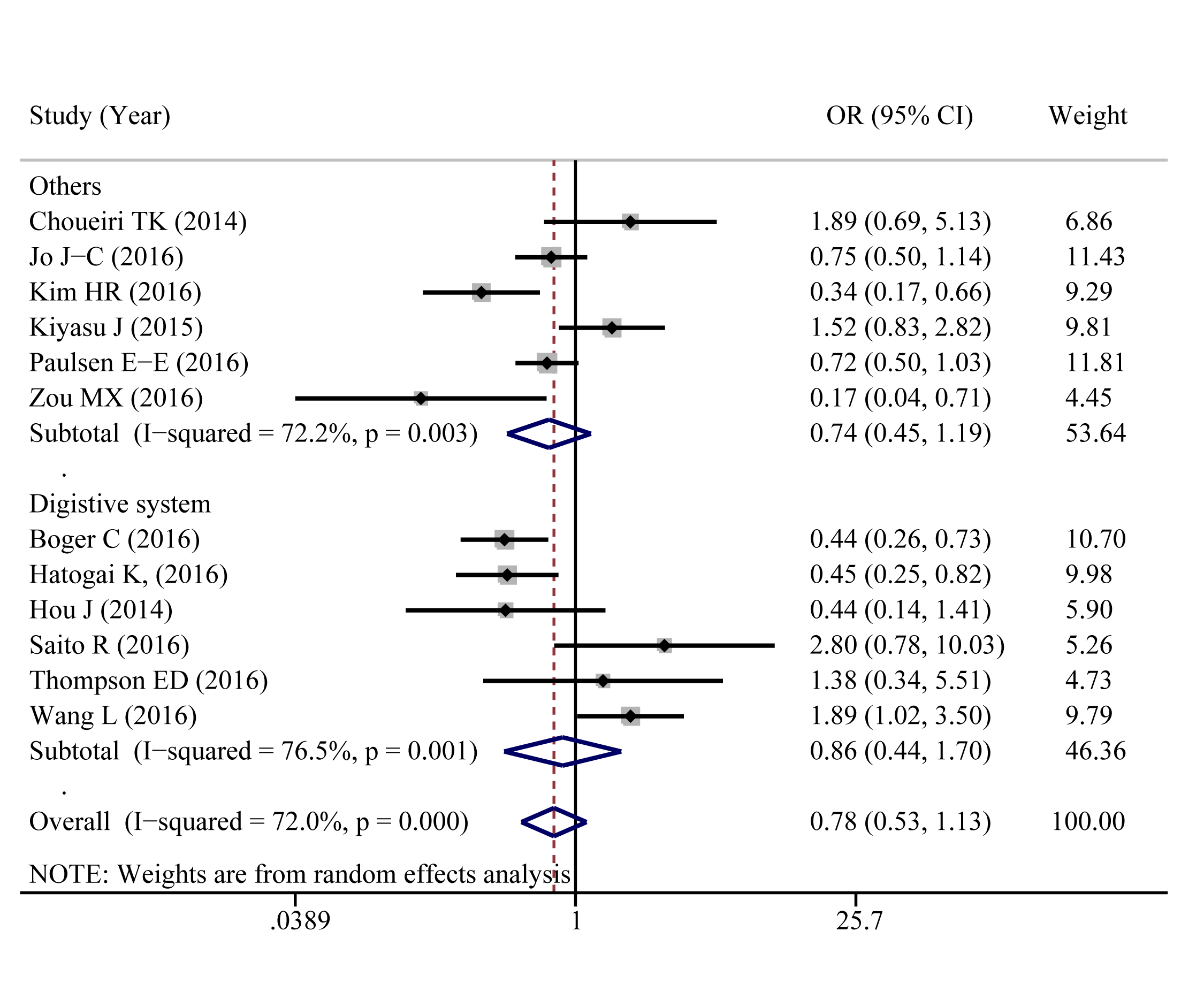

Supplement: S1 Fig — (TIF) [file pone.0176822.s001.tif]

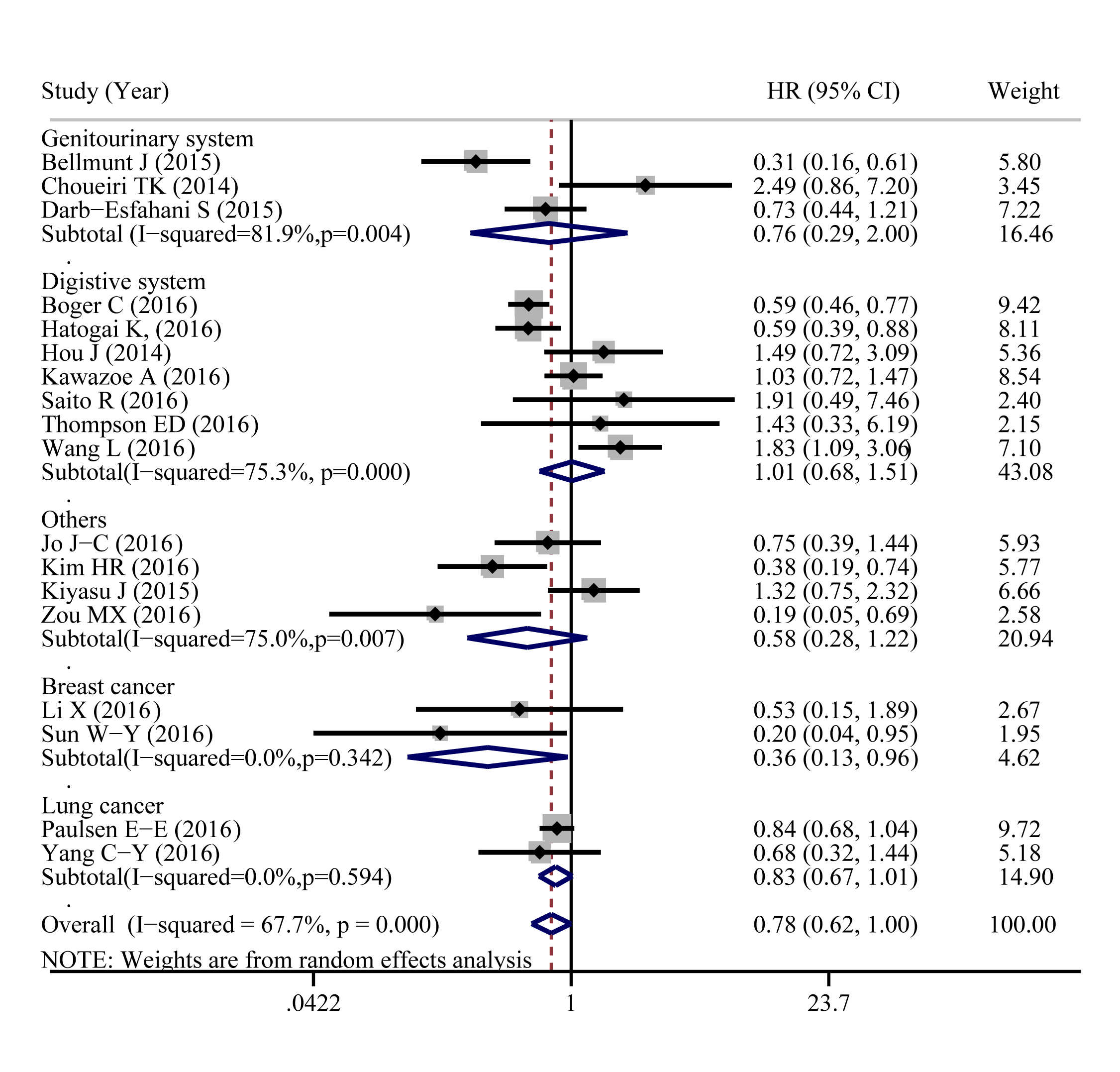

Supplement: S2 Fig — (TIF) [file pone.0176822.s002.tif]

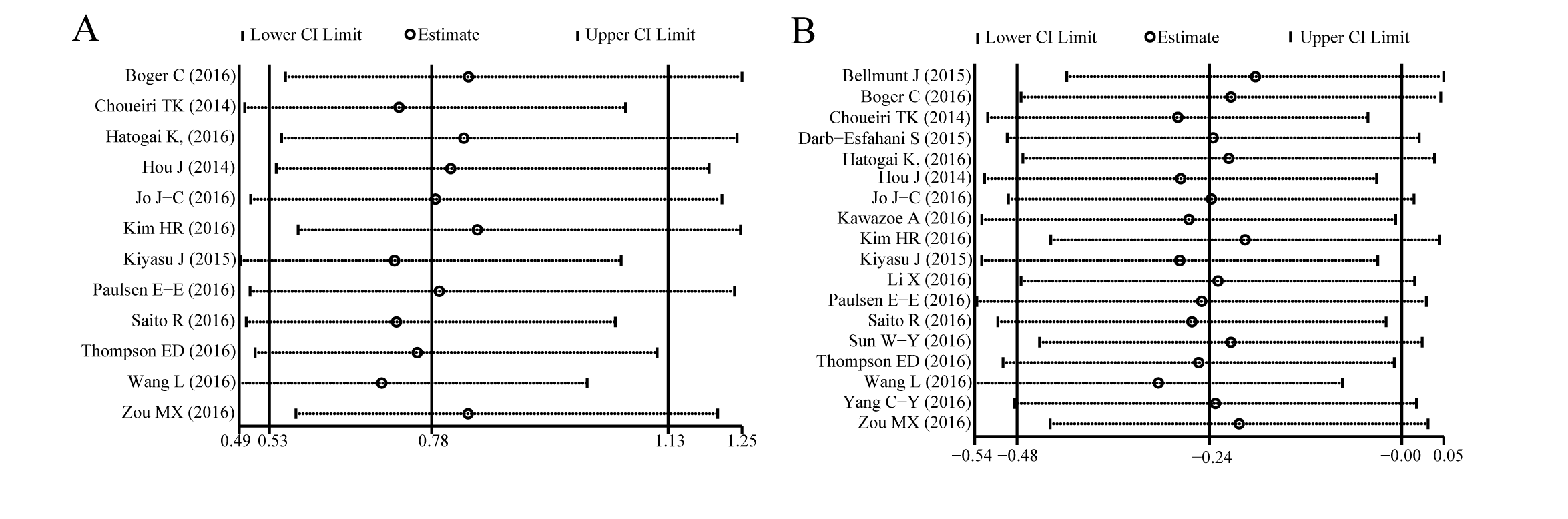

Supplement: S3 Fig — (A) Sensitivity analysis for 5-years OS (B) Sensitivity analysis for HR. (TIF) [file pone.0176822.s003.tif]
