# Supplementary material for: Prognostic value of PD-L1 expression in tumor infiltrating immune cells in cancers: A meta-analysis
Source: PLoS One. 2017 Apr 28;12(4):e0176822. doi: 10.1371/journal.pone.0176822 (PMC5409185; doi:10.1371/journal.pone.0176822)
Supplement: S2 File — (DOCX) [file pone.0176822.s005.docx]

**Full electronic search strategy in PUBMED**

((((("antigens, cd274"[MeSH Terms] OR ("antigens"[All Fields] AND "cd274"[All Fields]) OR "cd274 antigens"[All Fields] OR "programmed death ligand 1"[All Fields]) OR ("antigens, cd274"[MeSH Terms] OR ("antigens"[All Fields] AND "cd274"[All Fields]) OR "cd274 antigens"[All Fields] OR ("pd"[All Fields] AND "l1"[All Fields]) OR "pd l1"[All Fields]) OR ("antigens, cd274"[MeSH Terms] OR ("antigens"[All Fields] AND "cd274"[All Fields]) OR "cd274 antigens"[All Fields] OR ("b7"[All Fields] AND "h1"[All Fields]) OR "b7 h1"[All Fields]) OR ("antigens, cd274"[MeSH Terms] OR ("antigens"[All Fields] AND "cd274"[All Fields]) OR "cd274 antigens"[All Fields] OR "cd274"[All Fields])) AND (("lymphocytes, tumor-infiltrating"[MeSH Terms] OR ("lymphocytes"[All Fields] AND "tumor-infiltrating"[All Fields]) OR "tumor-infiltrating lymphocytes"[All Fields] OR ("tumor"[All Fields] AND "infiltrating"[All Fields] AND "lymphocyte"[All Fields]) OR "tumor infiltrating lymphocyte"[All Fields]) OR TIL[All Fields] OR (("tumour"[All Fields] OR "neoplasms"[MeSH Terms] OR "neoplasms"[All Fields] OR "tumor"[All Fields]) AND infiltrating[All Fields] AND immune[All Fields] AND ("cells"[MeSH Terms] OR "cells"[All Fields])) OR TIIC[All Fields] OR (("tumour"[All Fields] OR "neoplasms"[MeSH Terms] OR "neoplasms"[All Fields] OR "tumor"[All Fields]) AND infiltrating[All Fields] AND mononuclear[All Fields] AND ("cells"[MeSH Terms] OR "cells"[All Fields])) OR TIMC[All Fields] OR (("tumour"[All Fields] OR "neoplasms"[MeSH Terms] OR "neoplasms"[All Fields] OR "tumor"[All Fields]) AND stroma[All Fields]))) AND (("neoplasms"[MeSH Terms] OR "neoplasms"[All Fields] OR "cancer"[All Fields]) OR ("carcinoma"[MeSH Terms] OR "carcinoma"[All Fields]) OR ("tumour"[All Fields] OR "neoplasms"[MeSH Terms] OR "neoplasms"[All Fields] OR "tumor"[All Fields]))) AND (("prognosis"[MeSH Terms] OR "prognosis"[All Fields]) OR ("mortality"[Subheading] OR "mortality"[All Fields] OR "survival"[All Fields] OR "survival"[MeSH Terms]))) AND ("1900/01/01"[PDAT] : "2016/12/31"[PDAT])
